# A Tomato Spotted Wilt Virus S RNA-based Replicon System in Yeast

**DOI:** 10.1038/s41598-017-12687-8

**Published:** 2017-10-04

**Authors:** Kazuhiro Ishibashi, Eiko Matsumoto-Yokoyama, Masayuki Ishikawa

**Affiliations:** Plant and Microbial Research Unit, Division of Plant and Microbial Sciences, Institute of Agrobiological Sciences, National Agriculture and Food Research Organization, 2-1-2 Kannondai, Tsukuba, Ibaraki 305-8602 Japan

## Abstract

Tomato spotted wilt virus (TSWV) is a negative-strand RNA virus of the order *Bunyavirales*, family *Tospoviridae*, genus *Orthotospovirus*. TSWV infects a broad range of plant species, causing serious economic losses. Despite its agronomic importance, molecular biological understanding of TSWV has been limited, partly due to the lack of a reverse genetics system, which would enable genetic manipulation of the virus. Here, we report that RNA synthesis by TSWV RNA polymerase occurs in the yeast *Saccharomyces cerevisiae* using a segment of the TSWV genome, S RNA expressed from cloned cDNA, as a template. Viral nucleocapsid protein was required for RNA synthesis. Replacement of the protein-coding and intergenic regions of TSWV S RNA by a yellow fluorescent protein (YFP)-coding sequence drastically increased the accumulation of both sense and antisense strands of the RNA, showing that this RNA was replicated. Using this system, we revealed that efficient RNA synthesis by TSWV RNA polymerase in yeast requires the 5′-terminal 17-nt and 3′-terminal ~50-nt regions of the TSWV S cRNA (complementary RNA to the genomic RNA) template.

## Introduction

One of the most important achievements in RNA virus research is the development of reverse genetics systems which make possible the genetic manipulation of viruses^1^. Reverse genetics systems have greatly advanced the field of virus molecular biology. In positive-strand RNA viruses, infectious RNA can be transcribed from cloned viral cDNA. However, the situation is more complicated for negative-strand RNA viruses, because their genomic RNA forms a ribonucleoprotein complex with viral RNA polymerase and nucleocapsid proteins, and naked RNA is not infectious. Negative-strand RNA viruses are classified into two groups, those with segmented genomes and those with non-segmented genomes. Schnell *et al*. (1994) described the recovery of recombinant rabies virus, a non-segmented negative-strand RNA virus, from host cells transformed with plasmids expressing viral RNA and proteins^2^. This was the first report of a reverse genetics system for a negative-strand RNA virus. Similar approaches have been successfully applied to many animal negative-strand RNA viruses, including segmented negative-strand RNA viruses^3^; however, the reverse genetics systems of plant-infecting negative-strand RNA viruses have not been reported until very recently. At present, the only plant negative-strand RNA virus for which reverse genetics system is available is *Sonchus yellow net virus* (SYNV), a non-segmented negative-strand RNA virus^4,5^. Wang *et al*.^5^ developed a method to rescue SYNV from *Nicotiana benthamiana* leaves expressing three viral proteins and antigenomic RNA by Agrobacterium infiltration, and used mutant viruses generated through this method to uncover viral protein function^5^.

*Tomato spotted wilt virus* (TSWV) is a negative-strand RNA virus of the order *Bunyavirales*, family *Tospoviridae*, genus *Orthotospovirus*. TSWV infects more than 1,000 plant species and has spread worldwide, causing serious economic losses^6–8^. TSWV has three segmented genomic RNAs. L RNA is negative sense and encodes protein L (RNA polymerase). M and S RNAs are ambisense and encode protein NSm and glycoproteins G1 and G2, and proteins NSs and N (nucleocapsid protein), respectively. Functional analyses of these proteins using heterologous expression systems have revealed that NSs is an RNA silencing suppressor^9^ and NSm is a cell-to-cell movement protein^10^. NSs and NSm are also known as avirulence determinants for the resistance by pepper *TSW* gene and tomato *SW-5b* gene, respectively^11^. However, the lack of a reverse genetics system has been an obstacle to studying how viral genes or RNAs work in the infection process. Exceptionally, analyses of spontaneous knockout mutants and reassortants showed that NSs is involved in adult thrips infection and transmission^12^.

Budding yeast (*Saccharomyces cerevisiae*) is an excellent model organism for studying fundamental processes of eukaryotic cells. Yeast has also been used as a model host of many animal and plant RNA viruses, providing many insights into viral RNA replication^13^. In this study, we aimed to develop a TSWV replicon system in yeast, and to determine the sequence requirements for TSWV RNA replication.

## Results

### TSWV S vRNA synthesis using the S cRNA template in yeast

**Figure 1:**
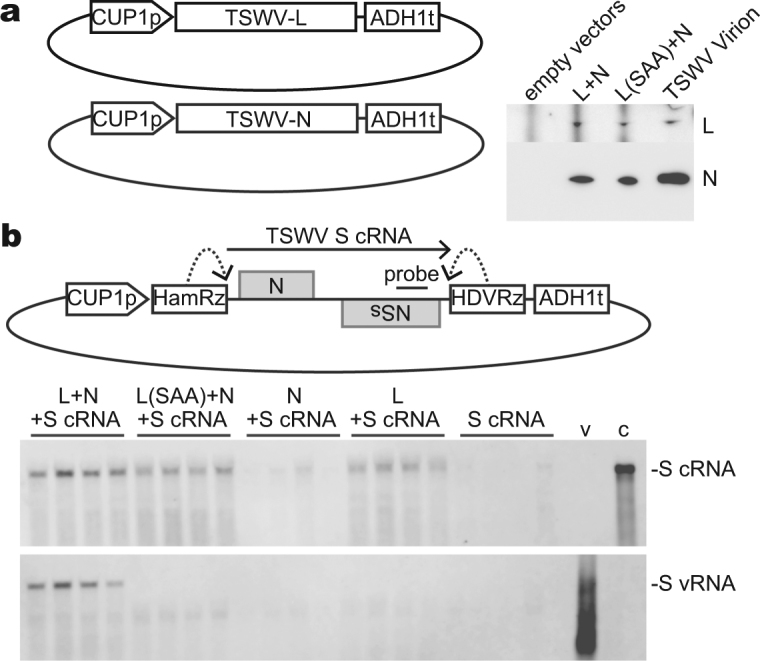
Tomato spotted wilt virus (TSWV) RNA synthesis in yeast. (**a**) Expression of L and N proteins in yeast. Protein accumulation following induction of expression was detected by immunoblotting. L(SAA) has mutations at the catalytic residues (SDD) of the L protein. Purified TSWV virion was loaded as a control. The original image is shown in Supplementary Fig. S1. (**b**) Expression of TSWV S RNA in yeast. Yeast was transformed with the indicated plasmids. After induction of expression, S cRNA and vRNA were detected by Northern blot hybridization. Lanes represent independent colonies. c and v are *in vitro* transcribed TSWV S cRNA and S vRNA, respectively. Note that prematurely terminated *in vitro* transcripts were detected only for S vRNA because of the position of probes.

Codon usage was optimized for the expression of TSWV L and N proteins in yeast. Codon-optimized L and N cDNAs were cloned under the control of the copper-inducible *CUP1* promotor, and introduced to yeast cells. Following induction of the *CUP1* promoter by CuSO_4_, we observed accumulation of L and N proteins (Fig. 1a, Supplementary Fig. S1). TSWV S cDNA was sandwiched between a hammerhead ribozyme and a hepatitis delta virus ribozyme and inserted downstream of the *CUP1* promoter such that TSWV S cRNA [i.e., complementary RNA to the TSWV S virion RNA (vRNA)] with precise terminal sequences was presumably generated after transcript self-cleavage. Yeast cells harboring the three plasmids were cultured for one day in the presence of CuSO_4_, and the accumulation of S cRNA and vRNA was examined by Northern blot hybridization. As negative controls, S cRNA was expressed alone, with either N or L protein, or with N protein and a mutant L protein whose SDD motif in the RNA polymerase catalytic site was changed to SAA. The mutant L(SAA) protein accumulated to a similar level as that of wild-type L protein in yeast (Fig. 1a). S cRNA was detected in all conditions, although the amounts varied among colonies (Fig. 1b). The S cRNA band demonstrated the same electrophoretic mobility as that of *in vitro*-transcribed full-length S cRNA, suggesting that ribozyme cleavage occurred efficiently. In the presence of L or L(SAA) protein, larger amounts of S cRNA accumulated (Fig. 1b), suggesting that L protein has a role in stabilization of the uncapped TSWV S cRNA independent of its RNA polymerase activity. In the presence of wild-type L and N proteins, we detected TSWV S vRNA (Fig. 1b). S vRNA was not detected in the absence of L or N protein or in the presence of the catalytic mutant L(SAA). These results indicate that S vRNA synthesis using the S cRNA template by TSWV RNA polymerase occurred in yeast. For clarity, we hereafter designate the RNA strand synthesized by pol II and its complementary strand as ‘expressed’ and ‘anti-expressed’ strands, respectively. In the above case, cRNA was the expressed strand and vRNA was the anti-expressed strand.

TSWV S cRNA and vRNA are templates for both replication and transcription^14^. The transcription products, NSs and N mRNA, are shorter than the replication products because transcription reaction terminates at an intergenic region to generate monocistronic mRNA. Although we observed several bands shorter than S cRNA and S vRNA, none were unique in cells expressing both L and N proteins (Fig. 1b). Thus, there is no evidence that TSWV NSs and N mRNA transcription occurred in yeast.

### The 5′- and 3′-terminal sequences of S RNA are necessary and sufficient for TSWV-guided RNA synthesis in yeast

**Figure 2:**
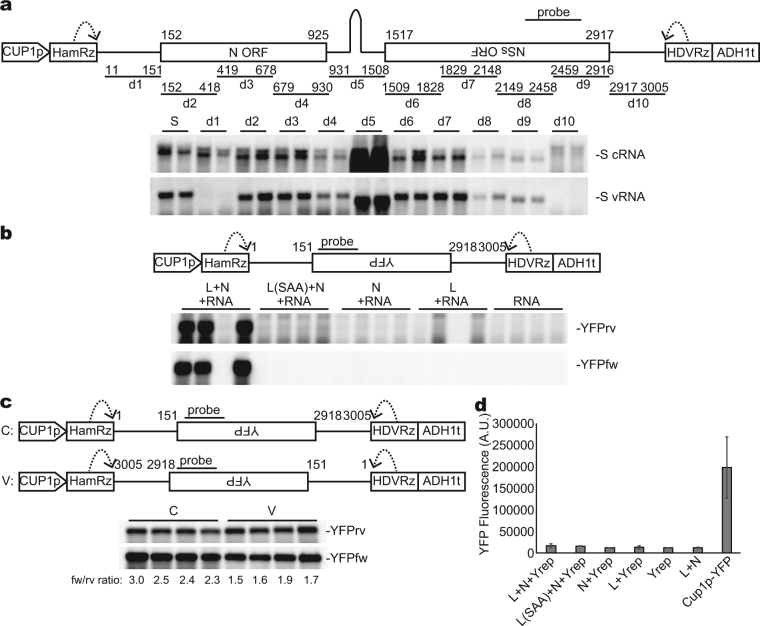
The 5′- and 3′-terminal UTRs of TSWV S cRNA are necessary and sufficient for TSWV RNA synthesis. (**a**) Deletion analysis of TSWV S RNA. Ten mutant RNAs lacking the indicated regions (d1–d10) were expressed in yeast with L and N proteins. S: unmodified TSWV S RNA. RNA was detected by Northern blot hybridization. Bands observed above the S cRNA and its derivatives likely represent uncleaved transcripts. Lanes represent independent colonies. (**b**) An RNA that contains 5′- and 3′-terminal UTRs of TSWV S cRNA can be replicated with the aid of both L and N proteins in yeast. In the panels marked YFPrv and YFPfw, expressed and anti-expressed RNA strands were detected, respectively. (**c**) The 3′ UTR of S cRNA has a stronger signal for complementary strand synthesis than the 3′ UTR of S vRNA. The UTRs were fused to the YFP sequence as shown in the figure and YFP-related RNAs were analyzed. Band intensity was quantified and the ratio of the intensity of the anti-expressed strand (YFPfw) to that of the expressed strand (YFPrv) is shown below the panel. (**d**) Quantification of YFP fluorescence in yeast cells expressing the YFP replicon (Yrep), N protein, and the L or L(SAA) mutant protein in the indicated combinations. As a positive control, YFP was expressed from the same vector as Yrep. Values are means of cultures from four independent colonies. Error bars represent standard deviation. A.U.: arbitrary unit showing YFP values normalized by optical density at 595 nm.

Using the system established above, we aimed to elucidate the sequence required for TSWV vRNA synthesis in yeast. A series of deletion mutants of S cRNA were expressed with the L and N proteins, and the accumulation of vRNA was examined by Northern blot hybridization. Among 10 deletion mutants, only d1 and d10, lacking the terminal untranslated regions (UTRs) corresponding to nucleotides 11–151 and 2917–3005 of TSWV S cRNA (3005 nucleotides), respectively, failed to accumulate vRNA (Fig. 2a), indicating that internal sequences including N and NSs open reading frames and the intergenic region are dispensable for vRNA synthesis. The d8 and d9 mutants lack a part of the sequence that hybridizes with the probes used in this study, and therefore the sensitivity for these mutant RNAs should be lower than that for other S-derived RNAs in this experiment. Remarkably, the d5 mutation, that excludes the intergenic region, enhanced the accumulation of cRNA (Fig. 2a), raising the possibility that the sequence in this region interferes with the expression of S cRNA. Note that the intergenic region of tospovirus S RNA, except for polygonum ringspot virus, forms a strong hairpin structure that could hamper the polymerase activity^15^.

To examine whether the terminal sequences of the cRNA template are sufficient for vRNA synthesis, the 5′ and 3′ UTRs of TSWV S cRNA (5′–151 nt and 3′–88 nt) were fused to a nonviral sequence complementary to the yellow fluorescent protein (YFP)-coding sequence. In the presence of L and N proteins, anti-expressed strand RNA was detected (YFPfw in Fig. 2b). In contrast, in yeast expressing L(SAA) and N proteins or lacking L or N proteins, or both, the anti-expressed strand RNA was not detected (YFPfw in Fig. 2b). The accumulation level of the expressed strand was also much higher in cells coexpressing L and N proteins than in negative-control cells (YFPrv in Fig. 2b), suggesting that this RNA was replicated and amplified in yeast. Hereafter, we designate this replicable RNA as the YFP replicon. When the 5′ and 3′ UTRs of the YFP replicon were replaced by respective UTRs of S vRNA (Fig. 2c, construct ‘V’), accumulation of the anti-expressed strand (YFPfw in Fig. 2c) relative to the expressed strand (YFPrv in Fig. 2c) became lower (1.5–1.9 vs. 2.3–3.0; Fig. 2c). This result suggests that the 3′ UTR of TSWV S cRNA has a stronger signal for initiation of RNA synthesis than the 3′ UTR of vRNA, which is consistent with the asymmetric replication of TSWV by which more vRNA than cRNA is produced in infected cells^16,17^. We did not observe a significant increase in YFP fluorescence in cells harboring the YFP replicon and L and N proteins over non-replicating negative controls (Fig. 2d). Thus, mRNA transcription from the YFP replicon did not occur in yeast at a detectable level, although we should take here into account that the YFP replicons lacks the intergenic region that acts as a transcriptional terminator and translational enhancer at the 3′ end of the TSWV mRNA^18^.

**Figure 3:**
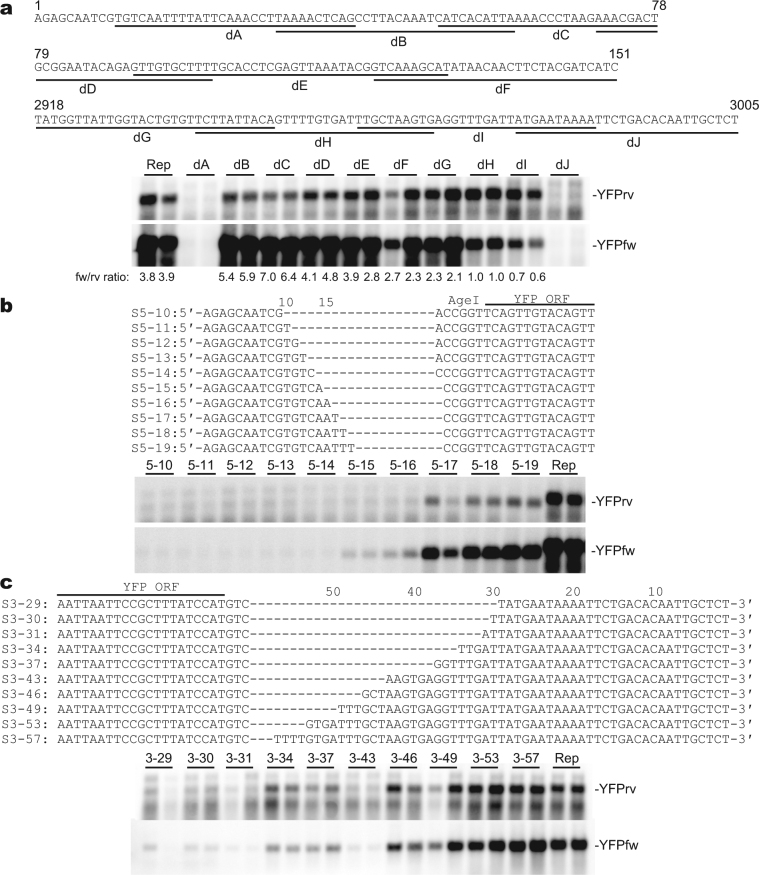
Exploration of the minimal sequence required for replication of the YFP replicon. (**a**) Deletion analysis of the UTRs of the YFP replicon. Six deletion mutants for the 5′ UTR and four deletion mutants for the 3′ UTR lacking the underlined sequences were expressed in yeast and RNA accumulation was detected by Northern blot hybridization. The ratio of the band intensity of the anti-expressed strand (YFPfw) to that of the expressed strand (YFPrv) is indicated below each lane. Rep: non-mutagenized YFP replicon. (**b**) Mapping of the replication element in the 5′ UTR of TSWV S cRNA. (**c**) Mapping of the replication element in the 3′ UTR of TSWV S cRNA.

Further deletions within the 5′ and 3′ UTRs of the YFP replicon showed the indispensability of the terminal regions in both UTRs (nucleotides 11–40 and 2978–3005 of TSWV S cRNA) for RNA synthesis (Fig. 3a). In contrast, deletions within internal regions of the UTRs affected RNA accumulation only when they were in the 3′ UTR of the template RNA: dH and dI mutants accumulated smaller amounts of YFPfw RNA whereas dB and dC mutants accumulated smaller amounts of YFPrv RNA (Fig. 3a). Thus, deleted sequences in these mutants may contribute to the initiation of RNA synthesis by TSWV RNA polymerase only when they are located in the 3′-terminal region.

To identify the minimal sequence required for TSWV RNA synthesis, we replaced each UTR of the YFP replicon by a series of short terminal sequences of TSWV S cRNA. YFPfw RNA was detectable when the 5′-terminal sequence was as short as 15 nt, and the accumulation level was comparable to the unmodified YFP replicon when the 5′-terminal sequence was 17 nt (Fig. 3b). The accumulation level of the YFPrv strand RNA was lower for the constructs 5–17, 5–18, and 5–19 than for the unmodified YFP replicon (Fig. 3b) as seen in the constructs dB and dC (Fig. 3a), again suggesting the presence of a polarity-specific enhancer of RNA synthesis. For the 3′ UTR, the minimum sequence was not clearly defined; the accumulation levels of both strands gradually increased as the 3′ UTR sequence became longer and reached the level of the unmodified YFP replicon around 50 nt (Fig. 3c).

## Discussion

Yeast has been used as a model host for many eukaryotic viruses^13^. The power of yeast genetics has greatly facilitated our understanding of viral replication. In this report, we demonstrated for the first time that a segment of plant negative-strand RNA virus genome expressed from cloned cDNA can be a template for viral RNA polymerase in yeast.

Using this system, we revealed that the terminal noncoding regions of TSWV S RNA are necessary and sufficient for replication. The importance of the terminal sequences for RNA synthesis has been reported in many viruses in the *Bunyavirales* ^19–23^, thus, this would appear to be a conserved property of these viruses. The 5′- and 3′-regions of the genomic RNAs of negative-strand RNA viruses show imperfect complementarity, and the genomic RNAs are proposed to form a panhandle structure through terminal base-paring. TSWV S RNA was predicted to form a panhandle structure of approximately 65–70 nt in length^24^. However, our results indicate that a sequence as short as 15–17 nt in the 5′ UTR was sufficient for complementary RNA synthesis whereas a longer sequence was required for the 3′ UTR. Thus, factors other than the formation of the panhandle structure are important for the terminal sequence of TSWV RNA in RNA synthesis. The crystal structure of the La Crosse orthobunyavirus L protein revealed that each of the 5′ and 3′ ends of the template RNA bind directly to different positions on the L protein, and the ~10-nt sequence from the 5′ end is likely important for the activation of the polymerase^25^. It is conceivable that the 17-nt region in the 5′ end of TSWV RNA also acts as an activator of the L protein. In contrast, a longer sequence in the 3′ UTR functions as a strand-specific enhancer of RNA replication. A longer sequence in the 5′ UTR of S cRNA also plays a role in vRNA-templated cRNA synthesis. Thus, the internal regions of the 3′ UTRs of both strands may have similar functions in RNA synthesis, possibly at the initiation step since RNA synthesis starts from the 3′ end of the template. S cRNA may contain a stronger 3′ enhancer of RNA replication than S vRNA to fulfill asymmetric replication.

In this yeast system, TSWV transcription was not clearly detected. Previous studies have indicated that purified TSWV virion has intrinsic RNA synthesis activity *in vitro* whereas transcription occurs only when a cell extract is supplemented^26,27^. The transcription-supporting activity copurified with translation elongation factor 1A from a tobacco cell extract^28^. Yeast may have an incompatible form of the transcription-supporting host factor. Thus, the yeast system may also be useful for studying the mechanism of TSWV mRNA transcription. Moreover, this system would facilitate the identification of host factors required for TSWV RNA replication. Considering the recent progress in new breeding techniques, the discovery of host factors may lead to future breeding of TSWV-resistant plants by disrupting the corresponding genes.

## Methods

### Plasmid Construction

TSWV was obtained from the NARO Genebank (MAFF 260050). TSWV S cDNA was amplified by reverse transcription-polymerase chain reaction (RT-PCR) from the viral RNA. A hammerhead ribozyme^29^ and hepatitis delta virus ribozyme^30^ sequences were added to the 5′- and 3′-end, respectively, by PCR. The nucleotide sequence of the cloned fragment is shown in Supplementary Fig. S2. Codons of the genes encoding TSWV L protein and N protein were optimized for expression in yeast by GENEART (Regensburg, Germany) and TaKaRa (Shiga, Japan), respectively. cDNA for the optimized TSWV L protein was cloned into the *CUP1* promoter and *ADH1* terminator-containing plasmid YCp22-CUP^31^ using SnaBI and SacI. The gene cassette of *CUP1* promoter, cloning site, and *ADH1* terminator from YCp22-CUP was transferred to YCplac111 and YEplac195 for cloning of the yeast-optimized cDNA for N protein and S RNA, respectively. The YFP-coding sequence from pTLPYFP^32^ was amplified and fused to the UTR sequences of S RNA to construct the YFP replicon (Supplementary Fig. S3). The YFP-coding sequence was also cloned into the YEplac195-based expression vector for fluorescence analyses. Deletion mutagenesis was performed by inverse PCR.

### Yeast Culture

A protease-deficient yeast strain BJ5465 (*MATa ura3–52 trp1 leu2D1 his3D200 pep4::HIS3 prb1D1.6R can1*) was used throughout the study. Precultured yeast cells harboring the plasmids were cultured in the presence of 0.2 mM CuSO_4_ at 30 °C for 5 h (for protein analysis) or 20 h (for RNA analysis). The cell pellet was homogenized by vortexing with glass beads and the extract was analyzed by Western blotting. RNA isolation from the cell pellet was performed using an RNeasy Mini Kit (QIAGEN, Hilden, Germany).

### RNA analyses

Total RNA from yeast cells was analyzed by Northern blot hybridization. Probe RNA was transcribed by T7 RNA polymerase in the presence of digoxigenin-11-UTP from the PCR fragments of TSWV S cDNA amplified using primers 5′-CCCGGGTAATACGACTCACTATAGTCAATACTAACGGAGTGAAAC-3′ and 5′-ATTGAAATTTGGCTTGAAACTGTAC-3′ to detect S cRNA, and 5′-CCCGGGTAATACGACTCACTATAGATTGAAATTTGGCTTGAAACT-3′ and 5′-GTCAATACTAACGGAGTGAAACATC-3′ to detect S vRNA (T7 promoter underlined). To detect the YFP replicon, we used digoxigenin-labeled oligodeoxyribonucleotides 5′-TTCGAAAGATCCCAACGAAAAGAGAGACCACATGGTCCTT-3′ or 5′-AAGGACCATGTGGTCTCTCTTTTCGTTGGGATCTTTCGAA-3′. Labeled probes on the hybridized Hybond-N + membranes (GE Healthcare) were detected with anti-digoxigenin-AP, Fab fragments and CDP-star (Roche).

### Antibodies

Anti-N protein antiserum was purchased from the Japan Plant Protection Association. Anti-L protein rabbit antiserum was raised against an *Escherichia coli*-expressed fragment of the TSWV L protein (amino acids 2295 to 2879, reading from the N-terminus).

### YFP fluorescence measurement

Yeast cells were cultured in the presence of 0.2 mM CuSO_4_ at 30 °C for one day. YFP fluorescence of the cultured yeast cells was measured by Tecan Infinite 200 Pro (Tecan, Männedorf, Switzerland) with 485-nm excitation and 535-nm emission wavelength filters. Values were normalized by the absorbance at 595 nm.

### Data Availability

The nucleotide sequences for codon-optimized L and N proteins were deposited to the DDBJ/EMBL/Genbank Database under accession numbers LC270910 and LC270911, respectively. All other datasets generated and/or analyzed during the current study are available from the corresponding author on reasonable request.

## Electronic supplementary material

Supplementary Figure S1, S2, S3
