# Supplementary material for: A Tomato Spotted Wilt Virus S RNA-based Replicon System in Yeast
Source: Sci Rep. 2017 Oct 4;7:12647. doi: 10.1038/s41598-017-12687-8 (PMC5627289; doi:10.1038/s41598-017-12687-8)
Supplement: Supplementary file 1 — Supplementary Figure S1, S2, S3 [file 41598_2017_12687_MOESM1_ESM.pdf]

Supplementary Information

A Tomato Spotted Wilt Virus S RNA-based Replicon System in Yeast

Kazuhiro Ishibashi, Eiko Matsumoto-Yokoyama, and Masayuki Ishikawa

Plant and Microbial Research Unit, Division of Plant and Microbial Sciences, Institute of Agrobiological Sciences, National Agriculture and Food Research Organization, 2-1-2 Kannondai, Tsukuba, Ibaraki 305-8602, Japan

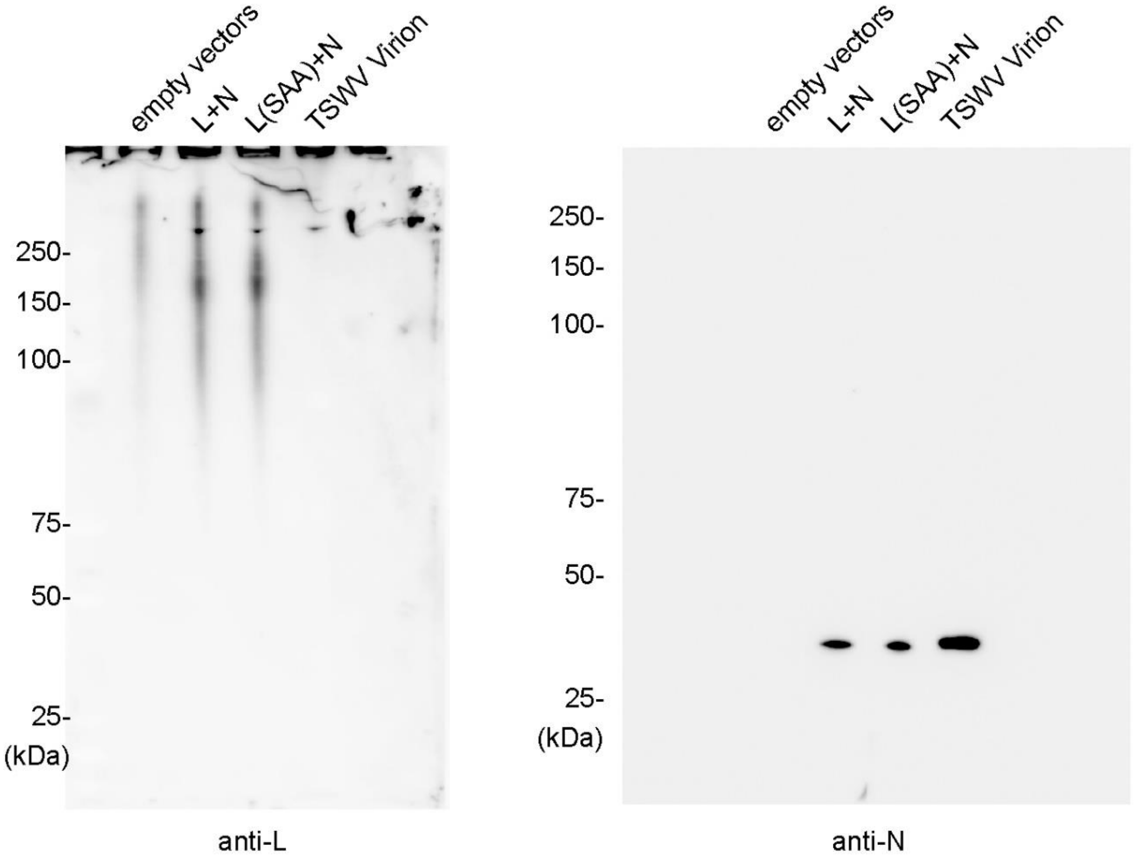

Supplementary Figure S1. The original image of Figure 1a. Positions of size markers (Precision Plus Protein Dual Color Standards, Bio-Rad) are indicated.

TACGTACGATTGCTCTCTGATGAGTCCGTGAGGACGAAACTATAGGAAAGGAATTCCTATAGTC
AGAGCAATCGTGTCAATTTTATTCAAACCTTAAAACTCAGCCTTACAAATCATCACATTAAAAC
CCTAAGAAACGACTGCGGAATACAGAGTTGTGCTTTTGCACCTCGAGTTAAATACGGTCAAAGC
ATATAACAACTTCTACGATCATCATGTCTAAGGTTAAGCTCACTAAGGAAAACATTGTTGCTTT
GTTGACACAAGGCAAAGACCTTGAATTTGAGGAAAATCAGAATCTGGTAGCATTCAACTTCAAG
ACTTTTTGTCTGGAAAACCTTGACCAGATCAAGAAGATGAGCATTATTTCATGTCTGACATTTC
TGAAGAATCGTCAGAGTATAATGAAGGTTATTAAGCAAAGTGATTTTACTTTTGGTAAAATTAC
CATAAAGAAAACTTCAGACAGGATTGGAGCCACTGACATGACCTTCAGAAGGCTTGATAGCTTA
ATCAGGGTCAGGCTTGTTGAGGAAACTGGAAATTCTGAGAATCTCAATACTATCAAATCTAAGA
TTGCTTCTCACCCTTTGATTCAAGCTTATGGATTACCTCTTGATGATGCGAAGTCTGTGAGGCT
TGCCATAATGCTGGGAGGTAGCTTACCTCTTATTGCTTCAGTTGACAGCTTTGAAATGATCAGT
GTTGTCTTGGCTATATATCAGGATGCAAAATACAAAGACCTCGGGATCGATCCAAAGAAGTATG
ACACAAAGGAAGCTTTAGGAAAAGTTTGCACTGTGCTGAAAAGCAAAGCATTTGAAATGAATGA
AGATCAGGTGAAGAAAGGGAAAGAGTATGCTGCTATACTTAGCTCCAGCAATCCTAATGCTAAA
GGAAGTGTTGCTATGGAACATTACAGTGAAACTCTTAACAAGTTCTATGAAATGTTCGGGGTTA
AAAAACAGGCAAAACTTGCAGAACTTGCTTAAAAGCAGCTGTAAGTTAAATTATAAAAGAACCT
ATAAATATATAAAGCTTTCTTTACCTTTATTGTTTGTGCTTGCTTAGTGTGTTAAATTTAAAAT
AAGTGTGTTTAATTAAAGTTTGCTTTCTGTGTGTTGTGCTTAATAAATAAACAAAATAACAAAA
ACAACAAAAACATAAAATAAAAATAAAAATAAAAATAAAAAATAAAAAATAAAAAATAAAAACA
AAAATAAAAATAAAATAAAATAAATAAAATAAAATAAAAATAAAAAATAAAAATAAAAAACAAA
AAACAAAAAACAAAAAACAAAAACAAAACCCAAATTTGGCCAAAATCGTCCCTTTCGGGATCTT
TTTTGGTTTTTTGTTTTTTAATTTTTTTGTTGTTTTTATTTTATTTTTTGATTTTTGTTTCATT
TTTTGATTTCTGTTTCATTCTGATTTTTATTTTTATTTTTATTTTTATTTTTGTTTTATAGTTT
CTATTAAACAGGAGAAATTCAAAAGAAATGACAAAACAGAAAAATAATTATAAGTAAAGAAAGA
AACAAACATAACATAATTAGAAAAAGCTGGACACGGCAAGATTATTTTGATCCTGAAGCATATG
CTTCTGAAATCTTAGATTCTTTCTTTTTGATCTCGCTTAAATCAAGCTTTAACAAAGATTTCGC
AACTGAAACAGATTGTGGAGAAATTTTAATTTCTCCCCTGGCAAAGTCTATTTTCCAGGAAGGG
ATTTGGATGCTGTCTAAGTAAGACATAGTTTGTGTGTTAGATGGAAGACATTCAAGTGTTTTTG
AAAGGAAATATTTCCTTTTGCAGATATCTTCACTGTAATTTAAGGTTCTTTCACCTAGATCTAA
CTTTCCTGGAGTTAGCTCATAGTTGTTCAAAGTGTAGATGATCACATCTTCTTGCAAGTTGTTG
CAAAGAACTTGTGCAAAAGATGTGTGAGTTTCAAGCAGAGCATGAACAATCCTCTGAGGATATG
AAGGATCATGAACAATGTTGTAAGGCTCCTTTAAATCAGAAAACATCATTGATAATTCAAAAGG
AGTTCTACATTTACGGATTGGGAGCTGATGCTTGCAAATAACAGAAATGTTTAAAGCTGTCTCA
ACGCTGTTATGATTTGGAATGCAAGCAATAGATAAATAAAATGTTTTGTTTGTTTCATCTCCTG
CAACTTTGAACAATTTCTGAATGGAAATCTGCTTCAAAACCTTTGGAACTCTTAGCCAGAGGCT
CAGCTTGAAATGAGAATCAGTGGAAGCTTTGACAAAAGCCTGAGAGTTAGGCATGATGTTGTTT

TCTGCTGACATAAGCAGAGATTTCACTGCAAGAGAATTCACAGTTCTGTTGTTGCTTTCAACTT
GATTGAAATTTGGCTTGAAACTGTACAGCCATTCATGGACATTTCTGTTAGGGGATAGAACATT
CACTTTGCCTAAAGCTTGATTGTAGCACATCTCGACCTTATAGGTATGCTCTTTAACACAAGAC
AAAGAGCCTTTGTTTGCAGCTTCAATGTATCTGTCATTGGGAATTATGTCTTTTTCTTGGAGCT
GGAATCGGTCTGTAATATCAGATCTGTTCACGGTAGATCCAATAGAGTGGAGCTGAGCAGGAGA
TAAAACTTTCAAATGACCTTGATGTTTCACTCCGTTAGTATTGACTGTATTTGAGCAAACAGAT
AGTGCCAGAACAGAGTCATCAATATTGATGCTAAAATCAATATCATCAAAAATAGGGATATACA
CATGCTGAGAAAGAATCTCTTCTTCTTCACAGGGAAGATTCCCTACCTTTGCAGTATAGCCAAA
GCTGCTTTTGCTTCTTGAATCAGAATACAGCTGGGTTTGAACTAGTGGAGAACCAGTACCAAGT
TCATGAATCCAGTAAGAATCTACAACTGCTTTACCAGATGCAGTTGATCCCCAGACTGAAGCTT
TTGTCTGAATGATCGACTCATAAACACTTGAAGACATTATGGTTATTGGTACTGTGTTCTTATT
ACAGTTTTGTGATTTGCTAAGTGAGGTTTGATTATGAATAAAATTCTGACACAATTGCTCTGGG
TCGGCATGGCATCTCCACCTCCTCGCGGTCCGACCTGGGCATCCGAAGGAGGACGCACGTCCAC
TCGGATGGCTAAGGGAGAGCCAGAGCTC

Supplementary Figure S2. The nucleotide sequence of the cloned fragment in TSWV S cRNA-expressing plasmid used in this study. Open reading frames for *N* and *NSs* genes are marked by light green and light blue, respectively. Restriction enzyme recognition sites (SnaBI and SacI) used for cloning are underlined. Hammerhead ribozyme and hepatitis delta virus ribozyme sequences are shaded.

TACGTACGATTGCTCTCTGATGAGTCCGTGAGGACGAAACTATAGGAAAGGAATTCCTATAGTC
AGAGCAATCGTGTCAATTTTATTCAAACCTTAAAACTCAGCCTTACAAATCATCACATTAAAAC
CCTAAGAAACGACTGCGGAATACAGAGTTGTGCTTTTGCACCTCGAGTTAAATACGGTCAAAGC
ATATAACAACTTCTACGATCATCACCGGTTCAGTTGTACAGTTCATCCATGCCATGTGTAATCC
CAGCAGCTGTTACAAACTCAAGAAGGACCATGTGGTCTCTCTTTTCGTTGGGATCTTTCGAAAG
CTTAGATTGATAGGACAGGTAATGGTTGTCTGGTAAAAGGACAGGGCCATCGCCAATTGGAGTA
TTTTGTTGATAATGGTCTGCTAGTTGAACGCCTCCATCTTCAATGTTGTGGCGGATCTTGAAGT
TCGCTTTGATTCCATTCTTTTGTTTGTCTGCCGTGATGTATACATTGTGTGAGTTATAGTTGTA
TTCCAATTTGTGTCCCAGAATGTTGCCATCTTCCTTGAAGTCAATACCTTTTAACTCGATTCTA
TTAACAAGGGTATCACCTTCAAACTTGACTTCAGCACGTGTCTTGTAGTTGCCGTCATCTTTGA
AGAAGATGGTCCTTTCCTGTACATAACCTTCGGGCATGGCACTCTTGAAAAAGTCATGCCGTTT
CATATGATCCGGGTATCTTGCAAAGCATTGAACACCATAGCCCAGAGTAGTGACTAGCGTTGGC
CATGGAACAGGCAGTTTGCCAGTAGTGCAGATGAGCTTCAGGGTAAGTTTTCCGTATGTTGCAT
CACCTTCACCCTCTCCACTGACAGAGAACTTGTGGCCGTTAACATCACCATCTAATTCAACAAG
AATTGGGACAACTCCAGTGAAGAGTTCTTCTCCTTTGCTGGTACCCAATTCGACCTTTCTCTTC
TTTTTTGGAGGCTCGGGAATTAATTCCGCTTTATCCATGTCGACTATGGTTATTGGTACTGTGT
TCTTATTACAGTTTTGTGATTTGCTAAGTGAGGTTTGATTATGAATAAAATTCTGACACAATTG
CTCTGGGTCGGCATGGCATCTCCACCTCCTCGCGGTCCGACCTGGGCATCCGAAGGAGGACGCA
CGTCCACTCGGATGGCTAAGGGAGAGCCAGAGCTC

Supplementary Figure S3. The nucleotide sequence of the YFP replicon. Open reading frames for YFP gene is marked by yellow. Restriction enzyme recognition sites (SnaBI, AgeI, SalI, and SacI) are underlined. Hammerhead ribozyme and hepatitis delta virus ribozyme sequences are shaded.
